# A One Health approach to antimicrobial resistance

**DOI:** 10.2478/abm-2022-0019

**Published:** 2022-08-31

**Authors:** Editorial Office of Asian Biomedicine

**Affiliations:** Faculty of Medicine, Chulalongkorn University, Bangkok 10330, Thailand

Antimicrobial resistance is an important problem resulting in part from a lack of antibiotic stewardship, which needs coordinated interventions designed to promote an optimal use of antimicrobial agents. Optimal use includes choosing appropriate drug regimens, optimal dosing, appropriate duration of treatment, and route of administration [1]. Antibiotics have been used not only in clinical settings, but also in agriculture and animal farming for food and animal proteins [2]. Antibiotic residues in farm products result in antibiotic-resistant bacteria that can be transmitted to humans via food chains, and can widely contaminate the environment via animal waste. The resistance resulting from clinical use and environmental contamination can complicate treatment of infections in humans. Resistance gives rise to fewer choices to treat infections in healthcare settings. In many instances, the choice of antibiotics for treatment of clinical infections have been based on empirical and broad-spectrum approaches, particularly when serious infections are suspected, while waiting for culture and drug susceptibilities. Healthcare providers do not always send clinical specimens for culture because they often consider that time to wait for analysis is too long, and the results do not always provide reliable information. The combination of time and effort required for proper antibiotic selection has sometimes led to indiscriminate broad-spectrum antibiotic use, and potential acquisition of antibiotic resistance [3].

The concept of the antibiotic resistome has been used to understand how resistance of bacteria, both pathological and nonpathological, emerges and evolves at the genetic level [4]. It includes all types of acquired and intrinsic antibiotic resistance genes. Mutations that result in resistance typically involve genes associated with essential functions of bacteria [5] and can be harmful to their survival [5,6,7] because they are less able to survive in a regular environment. This implies that the nature of antibiotic resistance is complex and may require a better conceptual framework to deal with the problem in addition to antibiotic stewardship. It is hoped that a more thorough understanding of antibiotic resistome can lead to an understanding of the resistance mechanism of microbes and their survival, which may lead to measures to reduce resistance and enhance new antibiotic discoveries [4].

In this issue, Loganathan and Nachimuthu report antibiotic resistance, biofilm forming ability, and clonal profiling of clinical isolates of *Staphylococcus aureus* from 2 regions in India [8]. They found that the prevalence of methicillin-resistant *S. aureus* is high in the regions studied, with many of the clinical isolates being multidrug resistant. They concluded that adopting “appropriate community-based preventive measures and establishing antimicrobial stewardship” is highly recommended to minimize the dissemination in antibiotic resistance.

“Appropriate community-based preventive measures” implies antimicrobial stewardship both in clinical and agricultural settings. This approach is crucial in addressing antibiotic resistance problems. The antibiotic resistance problem can reach a crisis when new antimicrobial medications have not kept pace with the rate of bacterial resistance. An additional approach, the global collection of resistance genes in clinical and environmental samples, or the antibiotic “resistome,” is a welcomed addition to antibiotic stewardship in clinical and environmental settings. Through bioinformatic analyses of modern-day sequencing, the antibiotic “resistome” approach may improve our understanding and control of antibiotic resistance genes or their transmission. Using a One Health approach in the collection and analysis of samples of specimens, the antibiotic “resistome” may help identify selective pressures affecting the emergence, transmission, and evolution of antibiotic resistance genes and antibiotic resistance. Meanwhile, management of patients with suspected or proven bacterial infection through empirical therapy, and tailoring them to the most specific antibiotics with appropriate dosing and duration, is needed. Strict antimicrobial oversight through prospective audit and feedback and/or preauthorization can be implemented based on facility-specific clinical practice guidelines for common infections [9].
